# Contribution of Oxidative Stress to HIF-1-Mediated Profibrotic Changes during the Kidney Damage

**DOI:** 10.1155/2021/6114132

**Published:** 2021-10-19

**Authors:** Hong Zhang, Renfeng Xu, Zhengchao Wang

**Affiliations:** Provincial Key Laboratory for Developmental Biology and Neurosciences, Key Laboratory of Optoelectronic Science and Technology for Medicine of Ministry of Education, College of Life Sciences, Fujian Normal University, Fuzhou 350007, China

## Abstract

Hypoxia and oxidative stress are the common causes of various types of kidney injury. During recent years, the studies on hypoxia inducible factor- (HIF-) 1 attract more and more attention, which can not only mediate hypoxia adaptation but also contribute to profibrotic changes. Through analyzing related literatures, we found that oxidative stress can regulate the expression and activity of HIF-1*α* through some signaling molecules, such as prolyl hydroxylase domain-containing protein (PHD), PI-3K, and microRNA. And oxidative stress can take part in inflammation, epithelial-mesenchymal transition, and extracellular matrix deposition mediated by HIF-1 via interacting with classical NF-*κ*B and TGF-*β* signaling pathways. Therefore, based on previous literatures, this review summarizes the contribution of oxidative stress to HIF-1-mediated profibrotic changes during the kidney damage, in order to further understand the role of oxidative stress in renal fibrosis.

## 1. Introduction

The balance of oxygen consumption and supply is essential for all mammalian organs, providing fuel for various physiological metabolic processes and maintaining homeostasis [1]. Kidney, an active metabolic organ, is a great need for oxygen. Thus, there is no doubt that the kidney is also susceptible to hypoxic damage.

There are increasing evidences shown that a variety of pathological factors such as hyperglycemia, hypersaline, hypertension, and infection can induce renal hypoxia and aggravate oxidative stress [2]. Meanwhile, it is demonstrated that acute kidney injury (AKI) and various chronic renal diseases (CKD) are associated with hypoxia and oxidative stress, which are more likely to develop into renal fibrosis eventually [3, 4]. Therefore, we have reasons to believe that hypoxia and oxidative stress may play an important role in the destruction of renal tissue, irreversible loss of kidney function, and the progression of renal fibrosis [5, 6].

Hypoxia inducible factors (HIFs), critical nuclear transcription factors, involved in maintaining O_2_ homeostasis were firstly discovered by Semenza in 1992, which have received extensive attention due to their significant role in cellular adaptation to hypoxia in recent years [7, 8]. Based on the difference of *α*-subunits, HIFs are divided into three subtypes, HIF-1, HIF-2, and HIF-3. The function of HIF-1 and HIF-2 is currently being intensively investigated. An increasing evidence finds that HIF-1 during kidney damage not only mediates hypoxia adaptation but also is associated with inflammation, epithelial-mesenchymal transition (EMT), and extracellular matrix (ECM) deposition, participating in the profibrotic changes [9–12]. And oxidative stress has been also reported to play an important role in this process [2]. HIF-2*α* plays a dominant role in erythropoietin production [13–15]. Schietke et al. also found that constitutional transgenic overexpression of HIF-2*α* in distal tubular cells in mice resulted in renal fibrosis [16]. Besides, a recent study has shown that SIRT1 can attenuate renal fibrosis by repressing HIF-2*α*. The effects of HIFs may be cell type and context dependent. HIF-2*α* may also be a candidate for studying renal fibrosis [17, 18]. However, HIF-3 is less well known. Other studies have shown that HIF-3*α* can act as a target gene of HIF-1 and negatively regulate the activity of HIF-1 and HIF-2 [19].

The present review is aimed at summarizing the profibrotic role and molecular regulation of HIF-1*α* on kidney damage, illustrating the interaction between HIF-1*α* and oxidative stress, and providing new insights for renal injury and aberrant tissue repair.

## 2. The Progression of Renal Fibrosis

Renal fibrosis is the final outcome of various kidney injuries and diseases. Although the reasons for fibrogenesis are diverse in different kidney diseases, the pathological process is similar. Usually, renal fibrosis can be artificially divided into four overlapping stages named as priming, activation, execution, and progression, respectively, according to different characteristics. Priming, the earliest stage of fibrogenesis, inflammatory cells can infiltrate into the kidney and be activated to secrete a variety of factors, such as chemokines, cytokines, and reactive oxygen species because of tissue damage. And then, secreted cytokines stimulate cells to undergo transformation and transdifferentiation to a myofibroblast phenotype, which expresses *α*-smooth muscle actin and produces a large amount of ECM proteins during the activation phase. In the stage of execution, ECM are accumulated in the interstitials and modified to resist proteolytic enzyme. The last stage of fibrosis is progression, which involves several types of kidney injuries, such as renal tubular atrophy and capillary rarefaction [20–22]. It is worth noting that the pathological process is irreversible once fibrosis emerges. Thus, it is crucial to understand the mechanism of renal fibrosis clearly and prevent fibrogenesis timely at the early stage of renal disease.

## 3. Oxidative Stress

Under normal physiological conditions, the body can produce a small amount of reactive oxygen species (ROS) [23]. And free radical scavenging enzymes and antioxidants maintain the balance of oxygen metabolism through activating transcription factors, regulating physiological active substances and inflammatory immunity, and promoting cell proliferation and differentiation, which has extensive physiological significance. However, once the levels between ROS and reactive nitrogen species (RNS) and antioxidant defense system cannot keep balance, oxidative stress appears [24, 25].

ROS is the main member inducing oxidative stress *in vivo*, mainly including superoxide anion and hydrogen peroxide. In cells, a large number of ROS are generated by the mitochondrial electron transport chain and cytochrome P450 family, and xanthine oxidoreductase, reduced nicotinamide adenine dinucleotide phosphate oxidase (NOX), nitric oxide synthase, and other catalytic enzymes greatly affect the generation of ROS [26]. RNS is a class of nitric oxide- (NO-) centered derivatives produced by the reaction of NO with ROS, including NO, nitrogen oxygen anion, nitrosothiols, and peroxynitrite. Excessive ROS and RNS can react with intracellular lipids, nucleic acids, and proteins, leading to lipid peroxidation, DNA oxidative damage, and intracellular protein denaturation, causing damage to cellular structure and function [27]. And oxidants can also act as signaling molecules to change intracellular signaling pathways and even gene expression [28]. In addition, oxidative modification can promote abnormal cell growth, inflammation, and other physiological processes [29, 30].

## 4. Hypoxia Inducible Factor-1

HIF-1 is a basic heterodimeric helix-loop-helix transcription factor and consists of an adjustable oxygen-sensitive *α*-subunit, HIF-*α*, and a constitutively expressed *β*-subunit, HIF-*β*. Hypoxia is the main regulation factor of physiologic HIF-1 expression. Besides, it is important to notice that HIF-1*α* overactivation can also be stimulated by some other mechanisms [31, 32].

### 4.1. Regulation of HIF-1 Hydrolysis

Oxygen-induced hydroxylation is one of the most important regulated pathways for HIF-*α*. Under normoxia, oxygen-dependent proline degradation domains on HIF-*α* can be hydroxylated by PHD [33]. Hydroxylated HIF-*α* can combinate with ubiquitin and be degraded by proteasome following the activation of von Hippel-Lindau tumor suppressor protein (pVHL), with the latter acting as a ubiquitin ligase to promote proteolysis of HIF-*α*. Factor inhibiting HIF (FIH) can also inhibit the transcriptional activity of HIF-*α* by hydroxylating asparaginic acid, while, under hypoxic conditions, the activity of PHD and FIH is suppressed, which further inhibits the hydroxylation and hydrolysis of HIF-*α*. Subsequently, the stabilized HIF-*α* dimerizes with HIF-*β* and translocates into the nucleus, activating a targeting gene [34].

### 4.2. HIF-1 Mediated Profibrotic Change

As a transcription factor, HIF-1 activation can regulate the expression of erythropoietin, vascular endothelial growth factor, endothelin-1, glucose transporters, and some other target genes, affecting erythropoiesis, angiogenesis, and energy metabolism, during which it governs the initial adaptation process to hypoxia, improves tissue oxygenation and cell survival, and to some extent offsets some harmful effects [9–11]. Although HIF-1 can reduce hypoxic-related damage under short-term hypoxia, increasing findings have suggested that HIF-1 can also play a significant role in the initiation and progression of kidney disease [12, 35–37]. Wang et al. demonstrated that chronic ischemia-induced overactivation of HIF-1*α* in the kidney mediates chronic renal damage [32]. Kimura et al. performed 5/6 nephrectomy on normal and VHL-knockout mice, finding that HIF-1 expression was stable and interstitial fibrosis was significantly severe in tubular epithelial cells of VHL-deleted mice [38]. And Baumann et al. found that knockout of the podocyte HIF-1*α* gene can prevent glomerular type I collagen accumulation and glomerulosclerosis [35]. Thus, HIF seems to promote the formation and development of fibrosis during kidney damage. Generally, renal fibrosis is characterized by inflammation, myofibroblast transformation, and extracellular matrix deposition [20, 21, 22]. Many researches have also demonstrated that HIF-1 may promote extracellular matrix remodeling to mediate renal fibrosis by inducing inflammation, EMT, collagen deposition, and ECM stiffening [39–41].

## 5. Contribution of Oxidative Stress to HIF-1-Mediated Profibrotic Changes

It has been described that during hypoxia, mitochondria increased the production of ROS, leading to inhibition of PHD activity and subsequent stabilization of HIF-1*α* protein [41]. Wang et al. have also demonstrated that ANG II stimulated H_2_O_2_ production, which inhibited PHD activity and thereby upregulated HIF-1*α* levels and consequently activated the tissue inhibitor of metalloproteinase, resulting in collagen I/III accumulation in cultured renal medullary interstitial cells [31]. PHD2 is the main subtype of renal PHD, mainly expressed in renal medulla. High salt intake initially increased renal tubular activity and decreased renal medullary oxygen level, thereby inhibiting PHD2 activity and activating HIF-1*α*-mediated adaptive genes. Proteins encoded by these genes produced medullary protective factors including NO, which in turn inhibited PHD2 [42]. Additional studies have suggested the involvement of PI-3K and ERK in NO-mediated HIF-1*α* accumulation [43, 44]. Others have also reported an increase in transcription of HIF-1*α* under hypoxia by ROS through induction of PI-3K/AKT and ERK phosphorylation [45, 46]. Oxidative factors can regulate the expression and activity of HIF-1 via PHD, ERK, and PI-3K/AKT.

While there is impaired PHD2 response to high salt in Dahl rats, increased oxidant stress might be one of the mechanisms. It is possible that high salt-induced oxidative stress induces PHD2 and thereby reduces HIF-1*α* levels in the renal medulla in Dahl S rats. Because of superoxide anion, it has been demonstrated to stimulate PHDs and thereby inhibit HIF-1*α* [47, 48]. Therefore, details of oxidative stress and PHD activity need to be clarified in future investigations. The relationship of oxidative stress and HIF-1 might be complex than our imagination. For example, it may be different in diverse animal models or distinct periods of diseases.

### 5.1. OS/NF-*κ*B/HIF-1 Signaling

Normally, inflammatory response is a process that the body resists to pathogen infection, which is controllable. However, if inflammatory response lasts a long time, it will cause damage and diseases to the body [49]. It is accepted that hypoxia is a common feature and an important cause of most inflammation. Studies have found that most kidney damage started with inflammation [50]. The nuclear factor-kappa B (NF-*κ*B) pathway is necessary for the expression of various proinflammatory factors under hypoxia, including TNF-*α*, IL-8, and IL-1*β* [51].

The study conducted by Jin and his colleagues has demonstrated that the oxidative stress/NF-*κ*B signal pathway contributed to the formation of unilateral ureteral obstruction renal interstitial fibrosis [52]. Under hypoxic environment, excessive ROS can activate the NF/*κ*B signaling pathway and then promote the expression of HIF-1*α* [53]. HIF-1*α* and NF-*κ*B signaling is highly dependent. Hypoxia and/or inflammation lead to increased NF-*κ*B and CCAAT/enhancer-binding protein delta (CEBPD) activity. CEBPD subsequently binds to the HIF-1*α* promoter and regulates HIF-1*α* signaling, thereby promoting inflammatory cell infiltration and inflammatory cytokine secretion in the renal tubulointerstitial region [54]. Zhao et al. showed that HIF-1*α* was upregulated in the kidneys of wild-type aristolochic acid nephropathy mice, accompanied by proximal tubular cell G2/M arrest and renal fibrosis [36]. Greijer and van der Wall have suggested that HIF-1 may inhibit the expression of cyclin-dependent kinase 1 and cyclins B1 and D1, leading to cell cycle G2/M arrest and promoting apoptosis in renal tubules [55]. Apoptosis induced by HIF-1 can release inflammatory mediators such as IL-1*β* and TNF-*α*, altering local renal microenvironment to trigger inflammation and fibrosis [56–58]. What is more, it has been shown that inflammatory cytokines can upregulate HIF-1*α* by MAPKp38 and via PI-3K/AKT phosphorylation [59].

Nevertheless, HIF can also inhibit renal inflammation by regulating Bcl-2 family genes, interacting with p53 or targeting mitochondrial enzymes to reduce tubular cell death [60, 61]. It can be seen that due to the complexity of the occurrence and development of inflammation, HIF-1*α* may play different roles in different stages of its development, which needs further study.

### 5.2. OS/HIF-1*α* /TGF-*β* Signaling

The activation of the NF/kB signaling pathway also plays an important role in the process of EMT and renal interstitial fibrosis in renal tubules [62]. EMT and ECM deposition are the key during renal aberrant trauma repair, which leads to fibrosis [63, 64]. During the process of EMT, proteins, such as e-cadherin, normally expressed by epithelial cells are lost and cell transdifferentiation markers, such as *α*-smooth muscle actin and fibroblast-specific protein 1, are obtained. An interesting finding shows that HIF-1*α* inhibited by short hairpin RNA can block the increasing expression of *α*-smooth muscle actin (SMA) in rats with a clipped kidney [32]. Higgins et al. found that activation of HIF-1 signaling in renal epithelial cells was associated with the development of chronic renal disease [64]. Experimental studies have shown that HIF-1 can activate various transcriptional regulators to promote mesenchymal transition by upregulating lysyl oxidase-like 2, B lymphoma Mo-MLV insertion region homolog1 (Bmi1), and Twist [12, 65, 66]. It has been demonstrated that HIF-1*α* stimulated collagen accumulation by activation of fibrogenic factors, such as connective tissue growth factor, plasminogen activator inhibitor, tissue inhibitor of metalloproteinase, and collagen proline and lysine hydroxylase [35, 67–69].

Transforming growth factor- (TGF-) *β* is considered to be the prototypical cytokine in renal fibrosis, which not only regulates the transformation of epithelial-mesenchymal cells to form myofibroblasts but also regulates the production and degradation of ECM [70, 71]. Zhou et al. indicate that ROS and HIF participate in hypoxia-induced TGF-*β* production [72]. HIF-1 accumulation can significantly enhance TGF-*β* expression [73–75]. TGF-*β* can upregulate gene expression of Nox4 NADPH oxidase or directly activate NADPH oxidase to generate ROS, which was reported to stabilize HIF-1*α* by decreasing PHD2 to reduce HIF-1*α* prolyl hydroxylation [76]. Das et al. found that the expression of NOX4 caused by TGF-*β* activation can be reduced by blocking Smad2 or Smad3, which suggested that TGF-*β*/Smad2/3 upregulated NOX4 and induced ROS generation, such as H_2_O_2_, which played an important role in the progression of renal fibrosis [76–78]. In TGF-*β*-treated renal tubular epithelial and mesangial cells, mammalian target of rapamycin complex-1 and Smad3 can also interact to increase the expression of HIF-1 and collagen [79]. Thus, TGF-*β* and ROS/HIF may form a feedback loop to maintain a prolonged signaling cascade initiated by either ROS/HIF or TGF-*β*.

### 5.3. miRNA/OS/HIF Signaling

The researches focused on the role of microRNA and HIF-1 during renal disease have also become more and more popular in recent years. MicroRNAs (miRNAs), small noncoding RNA molecules, can combine with 3′ untranslated regions of their target messenger RNA to inhibit their translation and thus regulate gene expression. A large number of studies show that miRNAs, such as miR217, miR23a, and miR-155, are closely related to the occurrence and development of renal fibrosis [80–84]. Recent studies have found that microRNA can regulate the expression or activity of HIF-1 by interfering with ROS production.

Increased miR-217 promotes inflammation and fibrosis in rats' glomerular mesangial cells cultured with high glucose through upregulating ROS, activating HIF-1 signaling pathway, and mediating cell apoptosis [80, 81]. miR-23a regulates cardiomyocyte apoptosis by suppressing the expression of MnSOD [82]. Li et al. also suggested that HIF-1 can induce exosome miR-23a expression, mediating the interaction between tubule epithelial cells and macrophages in tubule interstitial inflammation [83]. Xie et al. demonstrated that HIF-1*α* can increase the level of miR-155, thus promoting ETM and fibrosis both *in vivo* and *in vitro* [84], while miR-155-5p inhibitor treatment significantly decreased ROS generation and H_2_O_2_ concentration in HK-2 cells incubated with oxalate [85]. Therefore, we speculate that miR-155-5p mediated EMT by upregulating ROS, thereby activating HIF-1 signaling, which may form a vicious cycle. In addition, miR-21 in extracellular vesicles may induce EMT through enhancing HIF-1*α* expression, and caloric restriction alleviates aging-related fibrosis of the kidney through downregulation of miR-21 [86]. It has also been shown that miR-21 is induced by H_2_O_2_ in vascular smooth muscles [87]. miR-21 silencing enhanced mitochondrial function, which reduced mitochondrial ROS production and thus preserved tubular functions. It is possible that the interplay between miR-21 and ROS may lead to the activation of AKT and ERK pathways and contribute to miR-21 regulation of HIF-1*α* [88].

## 6. Conclusion

**Figure 1:**
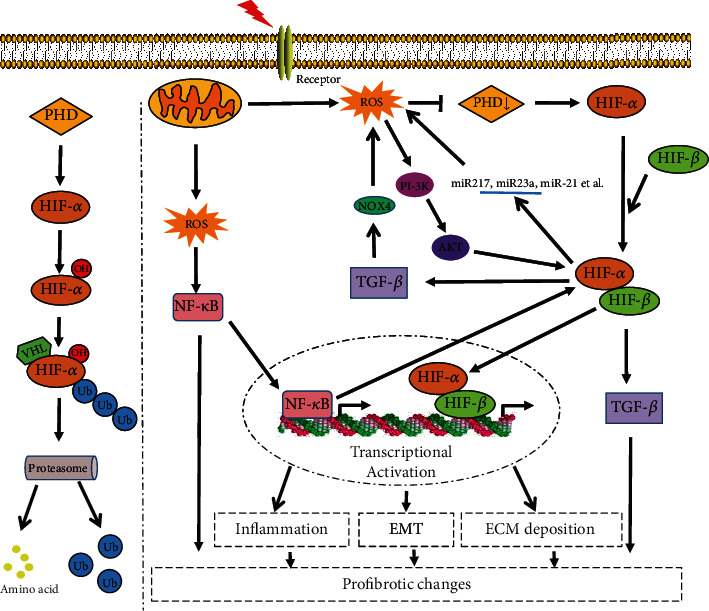
Contribution of oxidative stress to HIF-1-mediated profibrotic changes during the kidney damage. (1) Under normoxia, HIF-*α* can be hydroxylated by PHD. Hydroxylated HIF-*α* can combinate with ubiquitin and be degraded following the activation of VHL, (2) while, under stress conditions such as hypoxia or inflammation, the increased ROS can suppress the activity of PHD, which further inhibits the hydroxylation and hydrolysis of HIF-*α*. (3) Meanwhile, excessive ROS can activate NF/*κ*B signaling and then promote the expression of HIF-*α*. (4) Stabilized HIF-*α* dimerizes with HIF-*β* and translocates into the nucleus, activating a targeting gene. HIF-1 can promote apoptosis and lead to the release of inflammatory mediators such as IL-1*β* and TNF-*α*, triggering inflammation, while inflammation can aggravate hypoxia and oxidative stress further. Besides, HIF-1 may promote EMT and ECM deposition to mediate profibrotic changes by activating various transcriptional regulators and fibrogenic factors. (5) HIF-1 accumulation can also significantly enhance TGF-*β* expression. TGF-*β* can upregulate gene expression of Nox4 NADPH oxidase or directly activate NADPH oxidase to generate ROS, which may form a vicious cycle to lead to renal fibrosis. (6) In addition, HIF-1 can also regulate the expression of various microRNAs such as miR217, miR23a, and miR-21, then affecting the generation of ROS and promoting the development of fibrosis via activating PI-3K signaling.

With the aging of the social population, more and more patients now suffer from diabetes, hypertension, chronic kidney disease, and fibrosis, especially in developed countries [89–91]. Hypoxia and oxidative stress play an indispensable role in the occurrence and development of renal damage induced by these factors [92]. ROS accumulation during hypoxia promotes inflammation through activating NF-*κ*B and mediating crosstalk with HIF-1 signaling. Besides, ROS can stabilize HIF, inducing TGF-*β* gene expression. Elevated TGF-*β* levels sustain the ROS production, maintaining prolonged ROS/HIF/TGF-*β* signaling. The possible interaction between microRNA and HIF-1 may provide a sight for revealing the profibrotic changes of HIF-1. The crosstalk of HIF-1 with other classical intracellular fibrogenic signaling pathways may be necessary to amplify fibrotic pathological response (Figure 1).

However, the results on studying the role of HIF-1 in renal fibrosis seem to be much more complex. Kapitsinou et al. found that the stable expression of HIF can inhibit cell apoptosis and inflammatory response and significantly reduce AKI-related renal fibrosis [93]. In addition, HIF-1 has been found to contribute to the activation of forkhead box O3, leading to increased autophagy and reduced oxidative damage, thus playing a role in renal protection [94]. Inconsistent results may be caused due to diverse experimental conditions, nature and duration of animal models, and methods of manipulating HIF activity. It is worth noting that these harmful or protective mediators are not always easily distinguished. The overall effect depends on the intensity and duration of their expression.

## Data Availability

The original contributions presented in the study are included in the article. Further inquiries can be directed to the corresponding author.
